# Effects of *Eimeria acervulina* infection on the luminal and mucosal microbiota of the cecum and ileum in broiler chickens

**DOI:** 10.1038/s41598-024-61299-6

**Published:** 2024-05-10

**Authors:** Philip M. Campos, Katarzyna B. Miska, Mark C. Jenkins, Xianghe Yan, Monika Proszkowiec-Weglarz

**Affiliations:** 1 Oak Ridge Institute for Science and Education (ORISE), USDA-ARS Research Participation Program, Oak Ridge, TN USA; 2 USDA-ARS, NEA Bioinformatics, Beltsville, MD USA; 3 USDA-ARS, NEA, Beltsville Agricultural Research Center, Animal Biosciences and Biotechnology Laboratory, 10300 Baltimore Avenue, B-307, Rm. 335, BARC-East, Beltsville, MD 20705 USA; 4 USDA-ARS, NEA, Beltsville Agricultural Research Center, Animal Parasitic Diseases Laboratory, Beltsville, MD USA; 5 USDA-ARS, NEA, Beltsville Agricultural Research Center, Environmental Microbial and Food Safety Laboratory, Beltsville, MD USA

**Keywords:** Microbiome, Parasite host response

## Abstract

Coccidiosis, an intestinal disease caused by *Eimeria* parasites, is responsible for major losses in the poultry industry by impacting chicken health. The gut microbiota is associated with health factors, such as nutrient exchange and immune system modulation, requiring understanding on the effects of *Eimeria* infection on the gut microbiota. This study aimed to determine the effects of *Eimeria acervulina* infection on the luminal and mucosal microbiota of the cecum (CeL and CeM) and ileum (IlL and IlM) at multiple time points (days 3, 5, 7, 10, and 14) post-infection. *E. acervulina* infection decreased evenness in CeL microbiota at day 10, increased richness in CeM microbiota at day 3 before decreasing richness at day 14, and decreased richness in IlL microbiota from day 3 to 10. CeL, CeM, and IlL microbiota differed between infected and control birds based on beta diversity at varying time points. Infection reduced relative abundance of bacterial taxa and some predicted metabolic pathways known for short-chain fatty acid production in CeL, CeM, and IlL microbiota, but further understanding of metabolic function is required. Despite *E. acervulina* primarily targeting the duodenum, our findings demonstrate the infection can impact bacterial diversity and abundance in the cecal and ileal microbiota.

## Introduction

The parasitic disease coccidiosis is a considerable health and management challenge in the poultry industry. Infection by the apicomplexan protozoa *Eimeria* causes damage to epithelial cells within the gastrointestinal tract (GIT), which can lead to malabsorption, inflammation, and accumulation of mucus^1^. Consequently, weight gain depression, inefficient feed conversion, and reduced egg production may occur, and *Eimeria* lesions can predispose chickens to secondary infection by *Clostridium perfringens*, potentially resulting in mortality by necrotic enteritis^2^. Together, these factors have a significant economic impact on poultry production, leading to an estimated US $14 billion in annual losses worldwide^3^. *Eimeria acervulina*, along with *Eimeria maxima* and *Eimeria tenella*, are among the most commonly found *Eimeria* species in poultry farms^4^, each targeting different sites in the GIT. For instance, *E. acervulina* has a predilection for the duodenum and upper jejunum of the small intestine^5^. In the past, antimicrobials were relied upon to treat coccidiosis, however, modern legislation has resulted in a transition away from antimicrobials and antibiotics to greater usage of vaccines and drugs. Drug resistance is a concern in control of coccidiosis, which has inspired interest in alternative solutions such as probiotics, prebiotics, antioxidants, essential oils, and feed additives^6,7^.

Research towards these alternative solutions has required a greater understanding of the bacterial communities in the gut microbiota and the influence of infection on these communities. Due to the growing body of 16S rRNA amplicon sequencing studies, the importance of the gut microbiota on chicken health has been shown, with factors such as nutrient exchange, immune system modulation, digestive system physiology, and pathogen exclusion being associated with the gut microbiota^8,9^. The gut microbiota in chickens’ small and large intestines differ both in absolute bacterial counts and bacterial taxonomic composition. Microbiota of the ileum, duodenum, and jejunum tend to be similar, having low diversity and often containing *Lactobacillus*, *Enterococcus, Turcibacter, Clostridium *sensu stricto, and bacteria from the Clostridium XI cluster (family Peptostreptococcaceae, genus *Romboutsia*)^10^. In contrast, the cecal microbiota has considerably higher absolute counts and diversity, containing hundreds of bacterial species, most of which belong to the phyla Firmicutes and Bacteroidetes, while there is minor representation from the phyla Actinobacteria and Proteobacteria^10^.

In analyses on the luminal and mucosal microbiota of the duodenum and jejunum, *E. acervulina* infection had significant effects on alpha diversity (e.g. richness and evenness) and beta diversity (e.g. differing populations in infected and control), especially in the mucosal microbiota during and after the peak of infection^11^. Although *E. acervulina* does not target the cecum and ileum directly, a study has shown an *E. acervulina* infection can affect cecal microbiota diversity^12^, and it has been suggested that *E. acervulina* infection could affect cecal microbiota by increasing endogenous loss of proteins and undigested dietary proteins in the GIT^13,14^. While there are various studies investigating mixed *Eimeria* species infections on the cecum and/or ileum^13,15–17^, less is known on the individual effects of *E. acervulina*. The aim of this study was to determine the effect of *E. acervulina* on the luminal and mucosal microbiota of both the cecum and ileum at multiple time points during infection.

## Materials and methods

### Animal care and tissue sampling

All animal care procedures were approved by the Institutional Animal Care and Use Committee (IACUC, protocol #18-025) of the Beltsville Agricultural Research Center (BARC). This study was carried out in accordance with relevant rules and regulations and was performed and reported in accordance with ARRIVE guidelines (https://arriveguidelines.org/). The following animal care procedures are as described in greater detail elsewhere^11^. Ross 708 male broilers (288 birds, 1 day of age) were obtained from Longnecker’s Hatchery (Elizabethtown, PA) and placed into 1.00 m^2^ open-top wire brooder pens (25 chicks per pen). Birds were moved at 19 days of age into 72 finisher units (Alternative Designs, Siloam Springs, AR) with 4 birds per pen. A corn-soybean-based diet (approximately 24% crude protein in crumble format) and water were provided to chicks ad libitum for the duration of the study. One-half of the birds (144 birds) were infected (IF) with 1 × 10^5^ *E. acervulina* oocysts (USDA #12 isolate) in a volume of 1.0 mL per bird by oral gavage at 21 days of age, while the remaining 144 birds were sham-infected with water (control [C]). The resulting 36 pens of C birds and 36 pens of IF birds were placed in separate areas of the facility to prevent cross-transmission.

Of the 4 birds per pen, 1 bird closest to the pen’s average weight (to prevent sampling of birds with outlier weights) was euthanatized at day 0, 3, 5, 7, 10, and 14 post-infection (PI) via cervical dislocation. There were 6 pens (*n* = 6 replicates per treatment) for each time point, resulting in 36 C birds and 36 IF birds euthanatized. To evaluate infection, plasma carotenoid concentrations, body weight, and crypt depths were recorded at each time point. For luminal and mucosal microbiota sampling, a total of 48 of the 72 birds were randomly chosen as follows: 25 IF birds consisting of 5 replicates per day PI (3, 5, 7, 10, 14) and 23 C birds consisting of 5 replicates from day 0; 4 from each of day 3, 7, and 14; and 3 from each of day 5 and 10. The cecum and ileum were dissected, and the cecal contents (CeL), cecal epithelial scrapings (CeM), ileal contents (IlL), and ileal epithelial scrapings (IlM) were collected. The cecum was defined as paired blind sacs of the large intestine anterior to the cloaca (one sac sampled), and the ileum was defined as the part of the small intestine that is posterior to the Meckel’s diverticulum. Isolated specimens were snap frozen in liquid nitrogen and stored at − 80 °C until bacterial DNA isolation.

### Library preparation and sequencing

DNA extraction, library preparation, and sequencing were performed as described in Ref.^18^, utilizing a DNeasy PowerSoil kit (Qiagen, Valencia, CA), PCR primers targeting the V3-V4 region of the 16S rRNA gene, and the Illumina MiSeq platform (Illumina, Inc., San Diego, CA), respectively. The 16S rRNA gene sequences determined in this study were deposited in the NCBI Sequence Read Archive database (SRA accession no. PRJNA1066775).

### Bioinformatics and data analysis

As performed in Campos et al. ^11^, the bioinformatics platform Quantitative Insights Into Microbial Ecology 2 (QIIME 2) version 2021.4^19^ was used to analyze the CeL, CeM, IlL, and IlM microbiota. Demultiplexed, paired-end sequence data were denoised with DADA2^20^ via the q2-dada2 plugin, using a quality cutoff of 30 to determine truncation settings. For taxonomic classification, the SILVA database was chosen over the commonly used Greengenes database due to previous studies demonstrating SILVA’s larger database size and more recent updates to taxonomy, potentially improving interpretation of results^21,22^. The SILVA reference sequences and taxonomy were obtained from the QIIME 2 Data Resources page (https://docs.qiime2.org/2022.2/data-resources/) as pre-formatted files using RESCRIPt, a process used to reduce inconsistencies and improve processing by removing duplicate sequences that are assigned different taxonomies^23^. Reads were extracted from reference sequences using the V3-V4 region forward and reverse primers (5ʹ-end: CCTACGGGNGGCWGCAG and 3ʹ-end: GACTACHVGGGTATCTAATCC, respectively). A feature classifier was created via q2-feature-classifier^24^ fit-classifier-naive-bayes using SILVA version 138 99% operational taxonomic unit reference sequences and taxonomy^25^. Amplicon sequence variants (ASVs) from DADA2 were assigned taxonomy via the q2-feature-classifier classify-sklearn naïve Bayes taxonomy classifier. Mitochondria and chloroplasts were filtered and excluded from the feature table. All ASVs were aligned with MAFFT^26^ via q2-alignment and used to construct a phylogeny with fasttree2^27^ via q2-phylogeny. Using alpha rarefaction plots produced via q2-diversity and considering the number of samples retained in the dataset, rarefaction, or subsampling without replacement, was performed. The following sampling depths were applied for alpha and beta diversity analyses via q2-diversity: 14,132 for CeL, 19,242 for CeM, 24,469 for IlL, and 3816 for IlM.

Alpha diversity is a measure of species richness and/or evenness within an individual sample and was analyzed with four metrics: Shannon diversity index, observed features (ASVs), Faith’s phylogenetic diversity (Faith PD)^28^, and evenness. Observed features and Faith PD both measure richness, though Faith PD considers the phylogenetic differences in ASVs^28^. The non-parametric Kruskal–Wallis test was used to analyze differences in alpha diversity between treatment groups (the infection status and days post-infection). Beta diversity is a measure of distance between sample bacterial compositions and was measured using UniFrac distance metrics, which incorporate phylogenetic distances^29^. Unweighted UniFrac considers the presence and absence of ASVs in samples, while weighted UniFrac considers the abundance of ASVs^30^. To test for significance differences in groups based on UniFrac distances, the non-parametric permutational analysis of variance (PERMANOVA) test was used. To visualize UniFrac distances between groups and clustering of samples with similar microbiota, principal coordinates analysis (PCoA) was utilized. Visualizations for alpha diversity significance and beta diversity PCoA were produced in R 4.0.3^31^ using the packages QIIME2R 0.99.35^32^ to import QIIME 2 PCoA results and tidyverse 1.3.0^33^ for data wrangling with dplyr and graph production with ggplot2.

Differential bacterial abundance between IF and C birds was analyzed using the linear discriminant analysis effect size (LEfSe) algorithm^34^. Feature tables at the genus level were exported from QIIME 2 and analyzed at the Huttenhower Lab Galaxy web server (http://huttenhower.sph.harvard.edu/galaxy) using the default parameters. Phylogenetic Investigation of Communities by Reconstruction of Unobserved States 2 (PICRUSt2) version 2.4.2 software was used to predict functional abundances based on marker gene sequences^35^. The MetaCyc Metabolic Pathways Database^36^ was used to produce functional abundance data, and the data were analyzed and visualized using STAMP 2.1.3^37^ to determine biological relevance of features.

## Results

### Infection with *Eimeria acervulina*

Infection was evaluated in a prior study, with plasma carotenoid concentrations being significantly lower in IF birds on days 5 and 7 post-infection, body weight being significantly lower in IF birds overall, and crypt depths being increased in IF birds, indicating an observable *E. acervulina* infection^38^.

### Microbiota profiles

**Figure 1:**
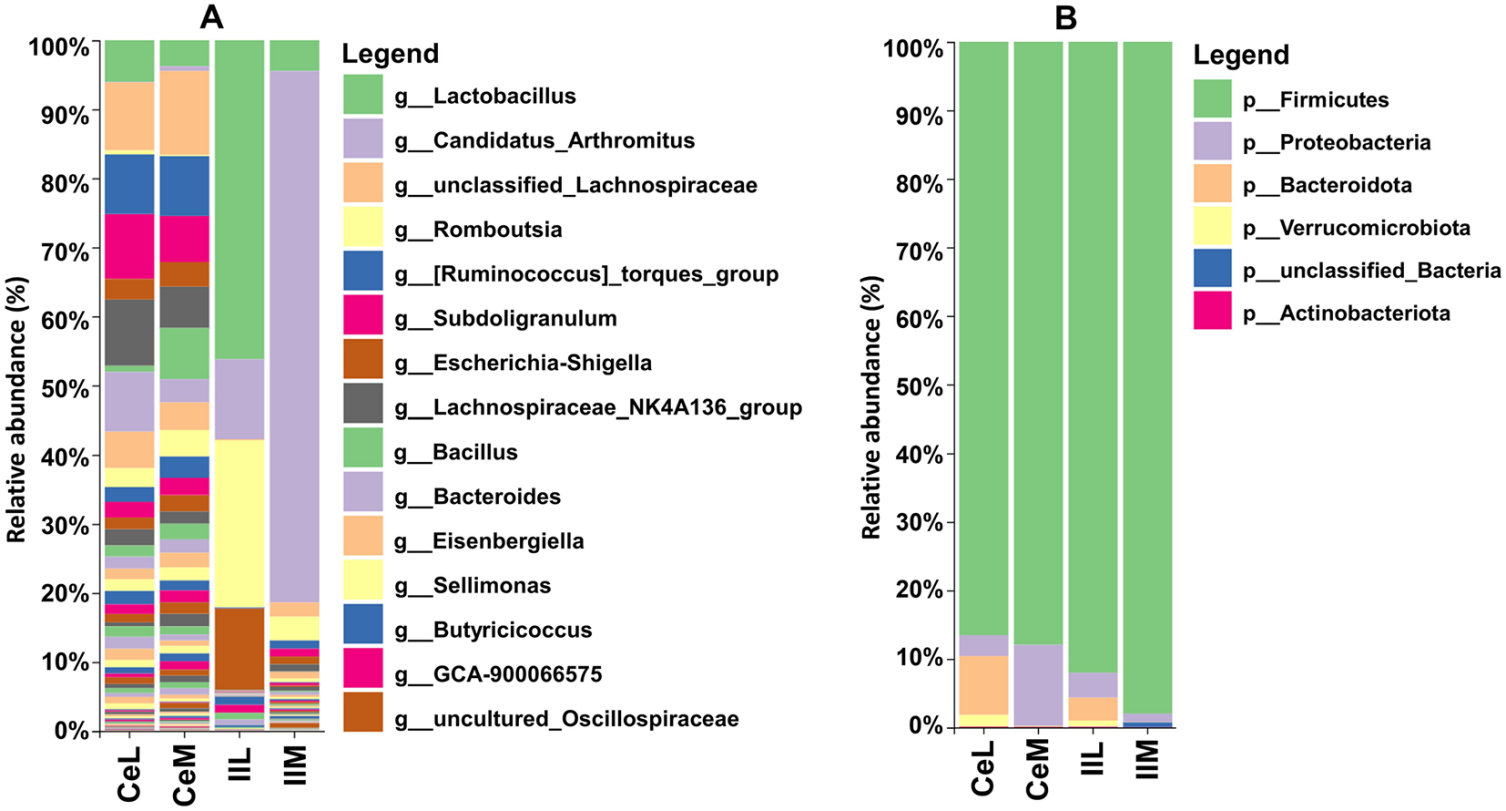
Summary of bacterial composition (relative abundance, %) in combined samples for CeL, CeM, IlL, and IlM microbiota for (**A**) genus level and (**B**) phylum level. In (**A**), the fifteen most common genera overall are listed in the legend, and in (**B**), the six most common phyla overall are listed.

Sequencing summaries for the four microbiota datasets (CeL, CeM, IlL, and IlM) are presented in Table 1. The five most abundant bacterial genera in CeL microbiota were unclassified Lachnospiraceae, *Lachnospiraceae NK4A136 group*, *Subdoligranulum*, [*Ruminococcus*] *torques group*, and *Bacteroides* (Fig. 1A). In CeM microbiota, the five most abundant genera were unclassified Lachnospiraceae, [*Ruminococcus*] *torques group*, *Bacillus*, *Subdoligranulum*, and *Lachnospiraceae NK4A136 group* (Fig. 1A). IlL microbiota were primarily comprised of *Lactobacillus*, *Romboutsia*, *Escherichia-Shigella*, *Candidatus Arthromitus*, and *Streptococcus* (Fig. 1A). Lastly, IlM microbiota were primarily comprised of *Candidatus Arthromitus*, *Lactobacillus*, *Romboutsia*, unclassified Lachnospiraceae, and *Subdoligranulum* (Fig. 1A). Broadly, the cecum was typically dominated by the phylum Firmicutes and contained smaller percentages of Bacteroidota, Proteobacteria, and Verrucomicrobiota (Fig. 1B). The ileum was also often dominated by Firmicutes, and Proteobacteria typically composed the remaining bacteria ranging from small to large percentages (< 1% to 72%) (Fig. 1B).

**Table 1:** Sequencing summary of four microbiota datasets processed in QIIME 2. QC = quality control via DADA2, ASVs = amplicon sequence variants. Reads after filtering indicates the number of reads after exclusion of mitochondria and chloroplasts.

	CeL	CeM	IlL	IlM
Number of samples	48	48	48	48
Raw reads	4,097,505	8,121,209	4,205,950	2,581,151
Reads after QC	2,622,668	4,845,239	3,099,871	1,818,413
Reads after filtering	2,622,101	4,845,101	2,957,530	1,817,219
Reads per sample (range)	9089–311,661	19,242–280,039	17,363–112,351	221–196,499
Mean reads per sample	54,627	100,940	61,615	37,859
Total number of ASVs	413	713	580	1356
ASV read length (range)	316–505	276–506	344–449	280–515
Mean ASV read length	427	407	435	401

### Alpha diversity

**Figure 2:**
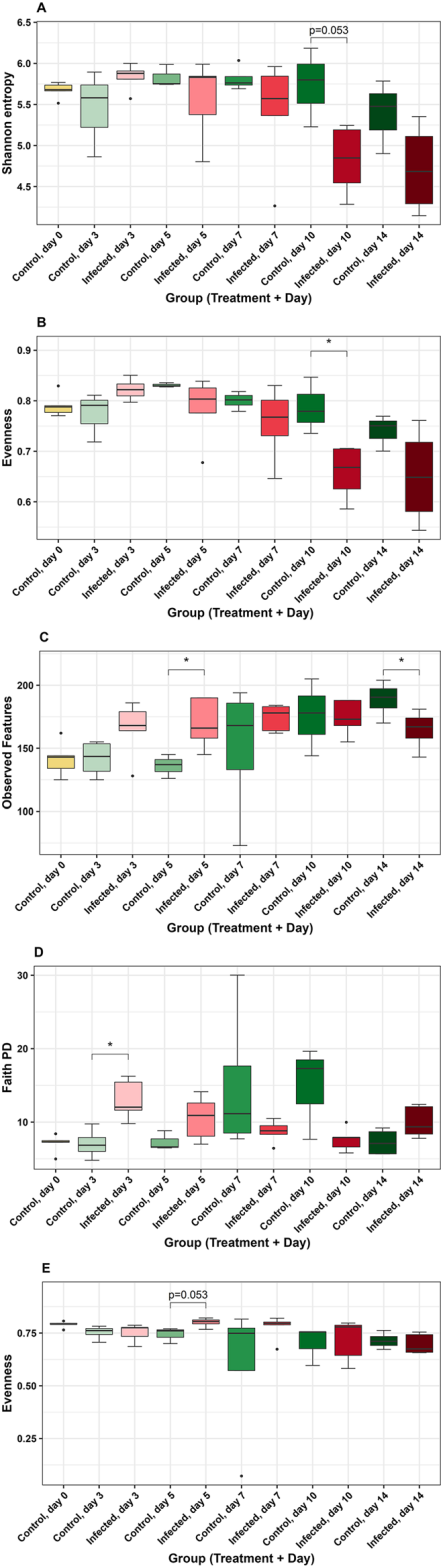
Comparisons of alpha diversity metrics in the cecum: (**A**) Shannon diversity in CeL, (**B**) evenness in CeL, (**C**) observed features (ASVs) in CeM, (**D**) Faith’s phylogenetic diversity (Faith PD) in CeM, and (**E**) evenness in CeM.

In CeL microbiota, Shannon diversity significantly differed based on group (infection status and time post-infection) (Kruskal–Wallis, H = 20.41, *P* = 0.02, Table S1). Pairwise comparisons showed there was a trend of lower Shannon diversity in IF birds compared to C birds on day 10 (H = 3.75, *P* = 0.053, Fig. 2A, Table S2A). Faith PD differed based on group overall (H = 18.97, *P* = 0.04, Table S1), however, there were no significant differences in comparisons between IF and C birds on particular days (*P* > 0.05, Table S2B). Evenness differed in groups overall (H = 26.58, *P* < 0.01, Table S1), with lower evenness in IF birds compared to C birds on day 10 (H = 5.00, *P* = 0.03, Fig. 2B, Table S2C). Observed features did not differ based on group (*P* > 0.05, Table S1).

In CeM microbiota, observed features differed in groups overall (H = 21.55, *P* = 0.02, Table S1), where observed features were higher in IF birds compared to C birds on day 5 (H = 4.46, *P* = 0.03, Fig. 2C, Table S3A), and there was a trend of lower observed features in IF birds on day 14 (H = 3.84, *P* = 0.05, Fig. 2C, Table S3A). Faith PD differed in groups overall (H = 21.19, *P* = 0.02, Table S1), with higher Faith PD in IF birds on day 3 (H = 6.00, *P* = 0.01, Fig. 2D, Table S3B). Evenness differed in groups overall (H = 21.39, *P* = 0.02, Table S1), and there was a trend of higher evenness on day 5 (H = 3.76, *P* = 0.053, Fig. 2E, Table S3C). Shannon diversity did not differ based on group (P > 0.05, Table S1).

**Figure 3:**
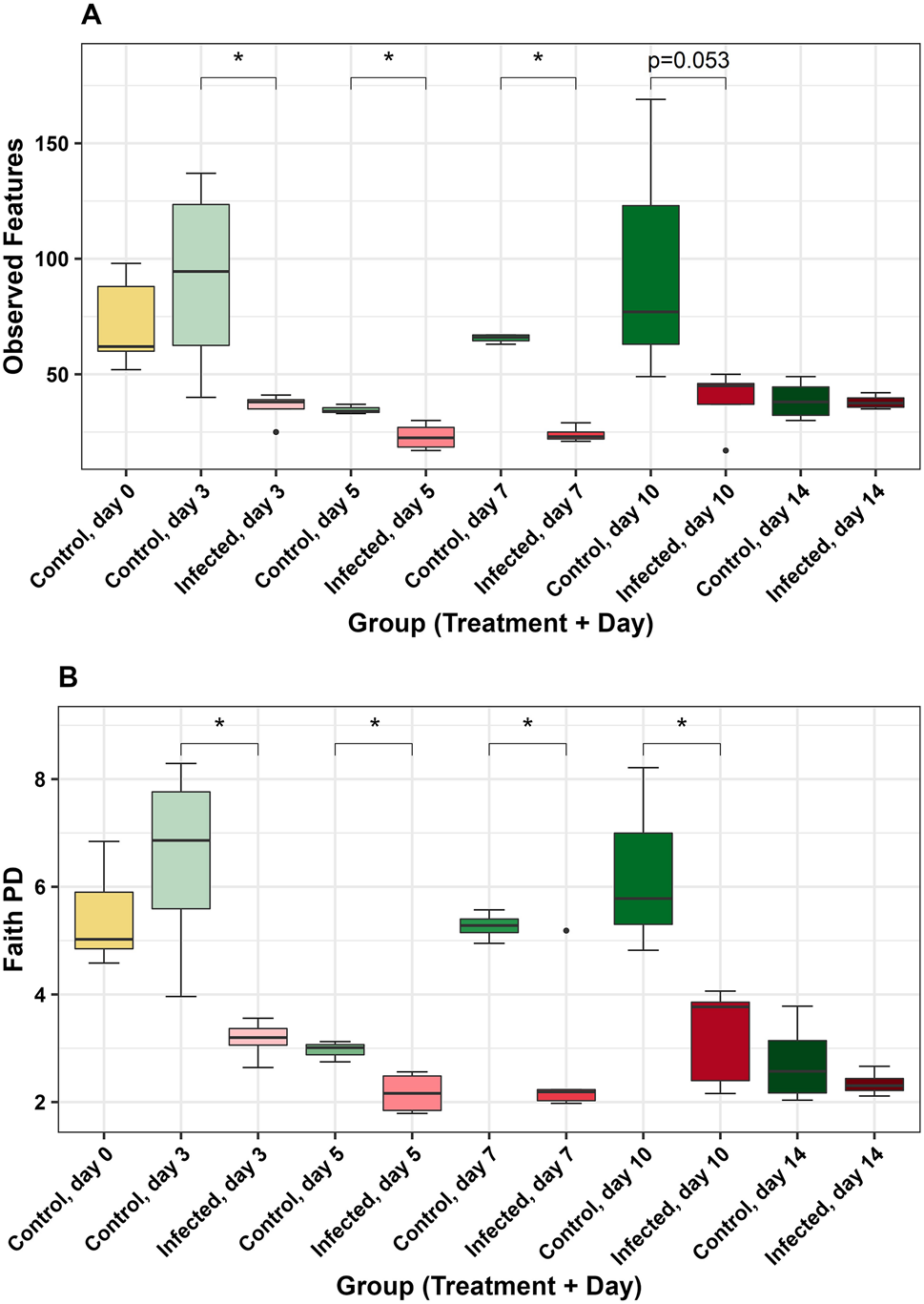
Comparisons of alpha diversity metrics in the ileal luminal microbiota: (**A**) observed features (ASVs), (**B**) Faith’s phylogenetic diversity (Faith PD).

In IlL microbiota, observed features differed in groups overall (H = 35.32, *P* < 0.01, Table S1), with lower observed features in IF birds compared to C birds on day 3 (H = 5.33, *P* = 0.03, Fig. 3A, Table S4A), day 5 (H = 4.50, *P* = 0.03, Fig. 3A, Table S4A), and day 7 (H = 6.05, *P* = 0.01, Fig. 3A, Table S4A). There was a trend of lower observed features in IF birds on day 10 (H = 3.76, *P* = 0.053, Fig. 3A, Table S4A). Faith PD differed in groups overall (H = 33.20, *P* < 0.01, Table S1), with lower Faith PD in IF birds on day 3 (H = 6.00, *P* = 0.01, Fig. 3B, Table S4B), day 5 (H = 4.50, *P* = 0.03, Fig. 3B, Table S4B), day 7 (H = 4.86, *P* = 0.03, Fig. 3B, Table S4B), and day 10 (H = 5.00, *P* = 0.03, Fig. 3B, Table S4B). There were no significant differences in groups for Shannon diversity and evenness (*P* > 0.05, Table S1).

In IlM microbiota, evenness differed in groups overall (H = 19.20, *P* = 0.04, Table S1), however, there were no significant differences between IF and C birds on a particular day (*P* > 0.05, Table S5A). There were no significant differences in groups for Shannon diversity, observed features, and Faith PD (*P* > 0.05, Table S1).

### Beta diversity

**Figure 4:**
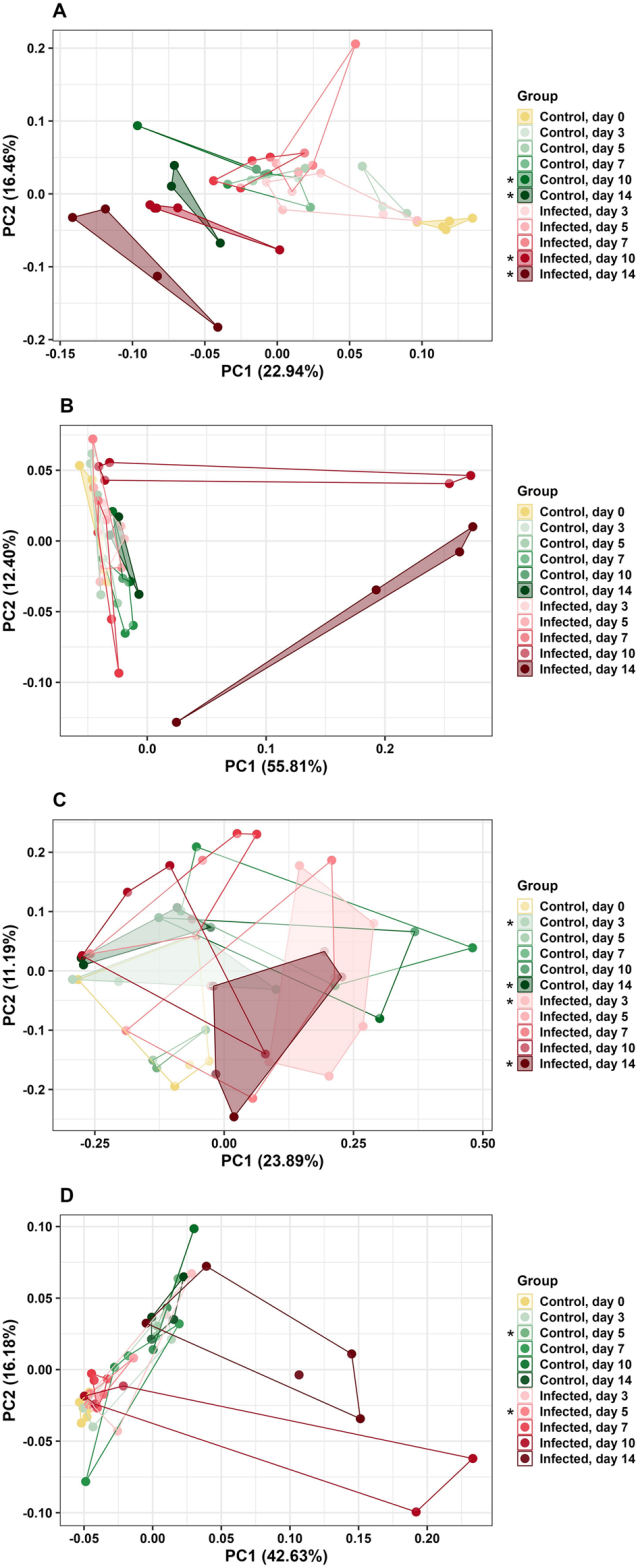
Principal coordinate analysis (PCoA) comparing *Eimeria acervulina*-infected birds and control birds at multiple time points based on (**A**) unweighted UniFrac distance matrix in CeL microbiota, (**B**) weighted UniFrac distance matrix in CeL microbiota, (**C**) unweighted UniFrac distance matrix in CeM microbiota, and (**D**) weighted UniFrac distance matrix in CeM microbiota. Stars (*) denote significant differences (*P* < 0.05) between microbiota of IF and C birds at the same time point.

CeL microbiota of different groups were considered distinct overall under unweighted UniFrac (PERMANOVA, pseudo-F = 3.06, *P* < 0.01, Fig. 4A, Table S1). Microbiota were distinct between IF and C birds on day 10 (pseudo-F = 3.28, *P* = 0.02, Fig. 4A, Table S2D) and day 14 (pseudo-F = 2.17, *P* = 0.05, Fig. 4A, Table S2D). Under weighted UniFrac, CeL microbiota were distinct based on groups overall (pseudo-F = 3.75, *P* < 0.01, Fig. 4B, Table S2E), and there was a trend of distinct microbiota between IF and C birds on day 14 (pseudo-F = 6.18, *P* = 0.057, Fig. 4B, Table S2E).

CeM microbiota of different groups were distinct overall under unweighted UniFrac (pseudo-F = 1.89, *P* < 0.01, Fig. 4C, Table S1), with distinct microbiota between IF and C birds on day 3 (pseudo-F = 2.15, *P* = 0.04, Fig. 4C, Table S3D) and day 14 (pseudo-F = 3.07, *P* = 0.01, Fig. 4C, Table S3D). Under weighted UniFrac, CeM microbiota were distinct based on groups overall (pseudo-F = 2.40, *P* < 0.01, Fig. 4D, Table S1), and IF and C birds had distinct microbiota on day 5 (pseudo-F = 7.60, *P* = 0.03, Fig. 4D, Table S3E).

**Figure 5:**
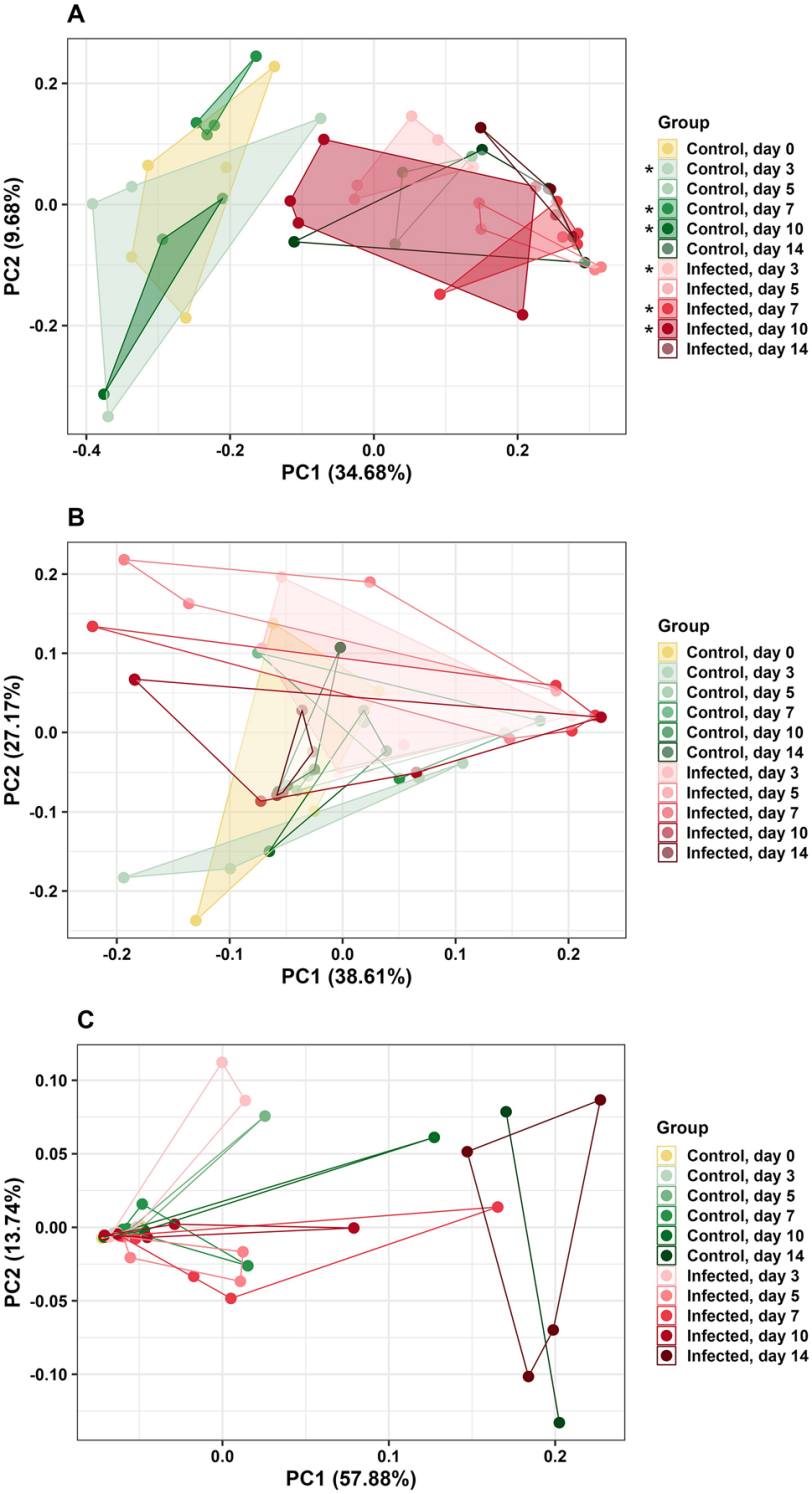
Principal coordinate analysis (PCoA) comparing *Eimeria acervulina*-infected birds and control birds at multiple time points based on (**A**) weighted UniFrac distance matrix in ileal luminal microbiota, (**B**) weighted UniFrac distance matrix in ileal luminal microbiota, and (**C**) weighted UniFrac distance matrix in ileal mucosal microbiota. Stars (*) denote significant differences (*P* < 0.05) between microbiota of IF and C birds at the same time point.

IlL microbiota of different groups were distinct overall under unweighted UniFrac (pseudo-F = 3.26, *P* < 0.01, Fig. 5A, Table S1), with IF and C birds having distinct microbiota on day 3 (pseudo-F = 4.04, *P* = 0.01, Fig. 5A, Table S4C), day 7 (pseudo-F = 6.36, *P* = 0.01, Fig. 5A, Table S4C), and day 10 (pseudo-F = 2.39, *P* = 0.03, Fig. 5A, Table S4C). There was a trend of distinct microbiota on day 5 (pseudo-F = 3.01, *P* = 0.055, Fig. 5A, Table S4C). Under weighted UniFrac, IlL microbiota were distinct based on groups overall (pseudo-F = 1.59, *P* = 0.04, Fig. 5B, Table S1), and there was a trend of distinct microbiota between IF and C birds on day 3 (pseudo-F = 2.57, *P* = 0.056, Fig. 5B, Table S4D).

IlM microbiota were not distinct under unweighted UniFrac (*P* > 0.05, Table S1). Under weighted UniFrac, IlM microbiota were distinct based on groups overall (pseudo-F = 3.90, *P* < 0.01, Table S1), however, microbiota were not distinct between IF and C birds on a particular day (*P* > 0.05, Fig. 5C, Table S5B).

### Differential abundance of bacterial taxa

**Figure 6:**
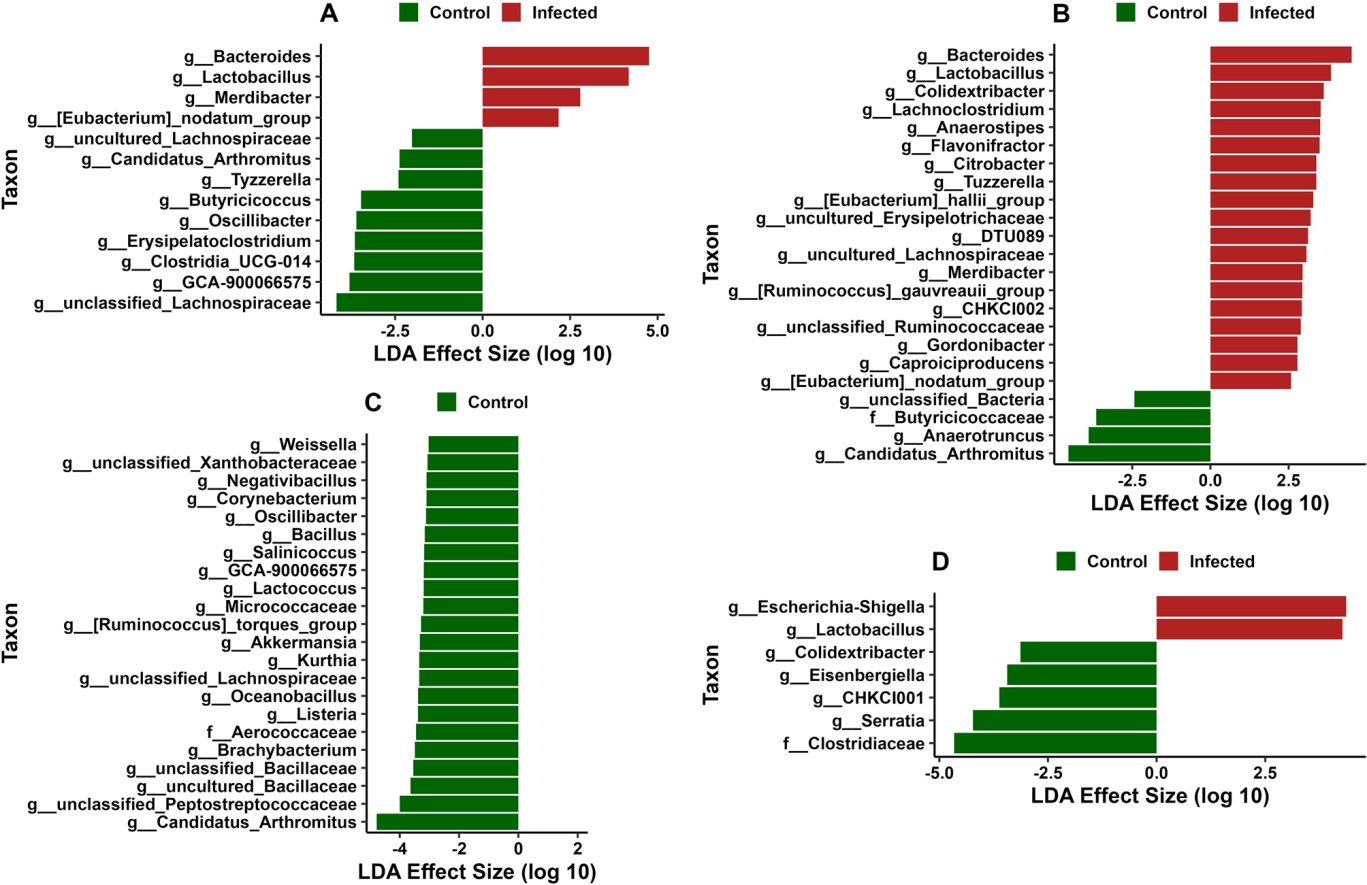
Differential bacterial abundance analysis using linear discriminant analysis effect size (LEfSe) in: (**A**) cecal luminal (CeL) microbiota, (**B**) cecal mucosal (CeM) microbiota, (**C**) ileal luminal (IlL) microbiota, and (**D**) ileal mucosal (IlM) microbiota. Positive effect size (red bars) indicates higher relative abundance in *Eimeria acervulina*-infected birds, while negative effect size (green bars) indicates higher relative abundance in control birds.

In CeL microbiota, the genera *Bacteroides* (Effect size (ES) = 4.76, *P* < 0.01), *Lactobacillus* (ES = 4.17, *P* = 0.02), *Merdibacter* (ES = 2.79, *P* = 0.02), and [*Eubacterium*] nodatum group (ES = 2.17, *P* = 0.03) were in greater relative abundance in IF birds compared to C birds, while 8 genera, including unclassified Lachnospiraceae (ES = 4.17, *P* = 0.03), *GCA-900066575* (ES = 3.80, *P* < 0.01), *Clostridia UCG-014* (ES = 3.67, *P* < 0.01), *Erysipelatoclostridium* (ES = 3.65, *P* < 0.01), and *Oscillibacter* (ES = 3.60, *P* = 0.02), were in greater relative abundance in C birds (Fig. 6A). In CeM microbiota, 19 genera (all *P* < 0.05), including *Bacteroides* (ES = 4.51, *P* < 0.01), *Lactobacillus* (ES = 3.85, *P* = 0.02), *Colidextribacter* (ES = 3.62, *P* = 0.04), *Lachnoclostridium* (ES = 3.53, *P* = 0.02), and *Anaerostipes* (ES = 3.51, *P* < 0.01), were in greater relative abundance in IF birds, while *Candidatus Arthromitus* (ES = 4.54, *P* < 0.01), *Anaerotruncus* (ES = 3.89, *P* = 0.02), an unclassified bacterium (ES = 2.43, *P* = 0.04), and the family Butyricicoccaceae (ES = 3.65, *P* = 0.04) were in greater relative abundance in C birds (Fig. 6B).

In IlL microbiota, no taxa had increased relative abundance in IF birds, while 48 genera (all *P* < 0.05) and the family Aerococcaceae (ES = 3.45, *P* = 0.01) were in greater relative abundance in C birds, including *Candidatus Arthromitus* (ES = 4.77, *P* = 0.02), unclassified Peptostreptococcaceae (ES = 4.00, *P* < 0.01), uncultured Bacillaceae (ES = 3.63, *P* < 0.01), unclassified Bacillaceae (ES = 3.54, *P* < 0.01), and *Brachybacterium* (ES = 3.49, *P* = 0.01) (Fig. 6C). In IlM microbiota, *Escherichia-Shigella* (ES = 4.36, *P* < 0.01) and *Lactobacillus* (ES = 4.28, *P* < 0.01) were in greater relative abundance in IF birds, while the family Clostridiaceae (ES = 4.66, *P* = 0.046) and genera *Serratia* (ES = 4.23, *P* = 0.04), *CHKCI001* (ES = 3.62, *P* = 0.03), *Eisenbergiella* (ES = 3.44, *P* = 0.01), and *Colidextribacter* (ES = 3.13, *P* < 0.01) were in greater relative abundance in C birds (Fig. 6D).

### Predicted functional abundances

**Figure 7:**
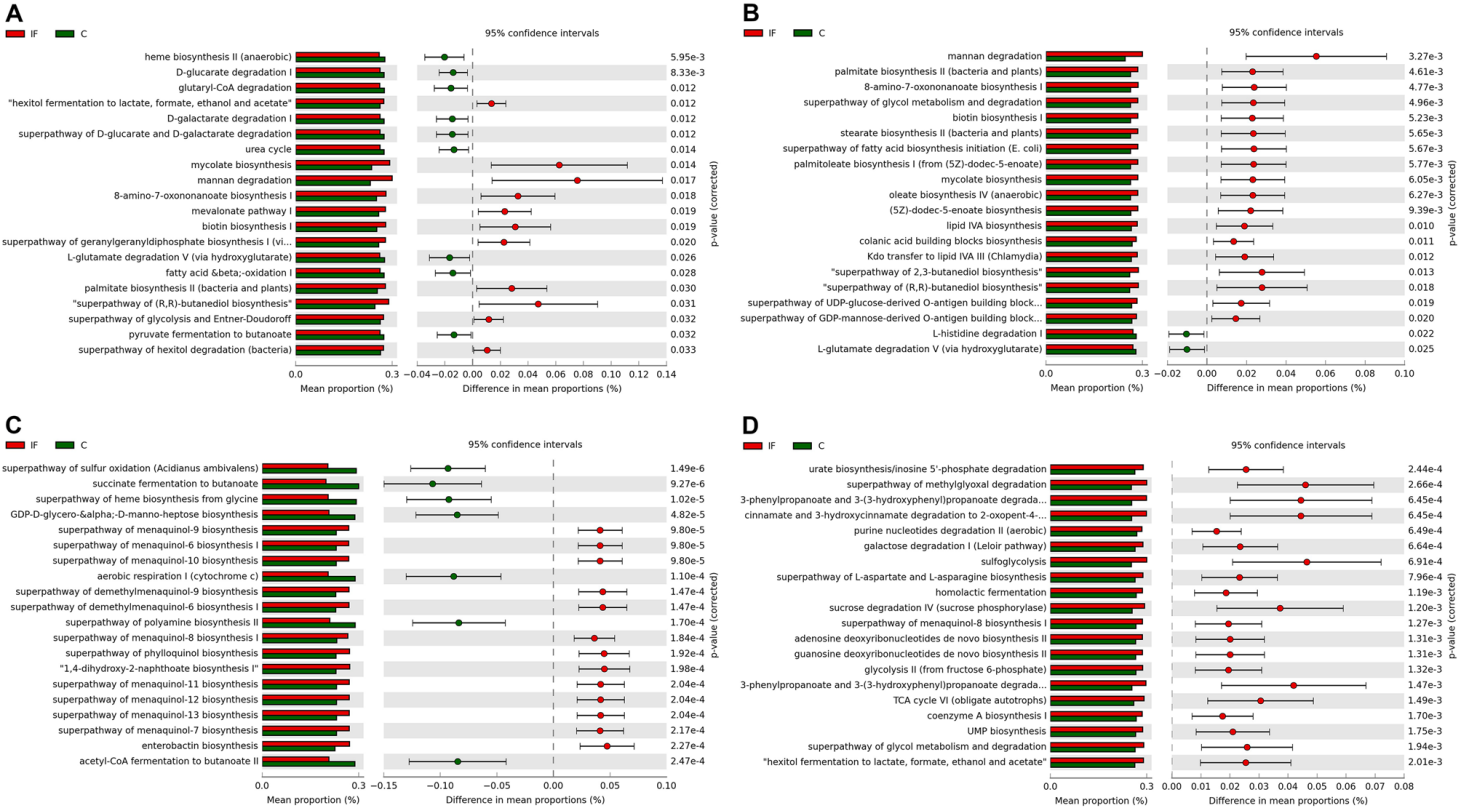
Effect of *Eimeria acervulina*-infection on differential mean proportion (%) of predicted MetaCyc pathways in the (**A**) cecal luminal (CeL), (**B**) cecal mucosal (CeM), (**C**) ileal luminal (IlL), and (**D**) ileal mucosal (IlM) bacterial populations.

In CeL microbiota, genes for 18 predicted MetaCyc pathways were in greater relative abundance in IF birds compared to C birds (all *P* < 0.05), including “hexitol fermentation to lactate, formate, ethanol, and acetate” (*P* = 0.01), mycolate biosynthesis (*P* = 0.01), mannan degradation (*P* = 0.02), 8-amino-7-oxononanoate biosynthesis I (*P* = 0.02), and mevalonate pathway I (*P* = 0.02), while 11 pathways were predicted to be in greater relative abundance in C birds (all *P* < 0.05), including heme biosynthesis II (anaerobic) (*P* < 0.01), d-glucarate degradation I (*P* < 0.01), glutaryl-CoA degradation (*P* = 0.01), d-galactarate degradation I (*P* = 0.01), and urea cycle (*P* = 0.01) (top 20 significant pathways in Fig. 7A). In CeM microbiota, 24 pathways were predicted to be in greater relative abundance in IF birds (all *P* < 0.05), including mannan degradation (*P* < 0.01), palmitate biosynthesis II (bacteria and plants) (*P* < 0.01), 8-amino-7-oxononanoate biosynthesis I (*P* < 0.01), superpathway of glycol metabolism and degradation (*P* < 0.01), and biotin biosynthesis I (*P* < 0.01), while 4 pathways were predicted to be in greater relative abundance in C birds, including l-histidine degradation I (*P* = 0.02), and l-glutamate degradation V (via hydroxyglutarate) (*P* = 0.03) (Fig. 7B).

In IlL microbiota, 115 pathways were predicted to be in greater relative abundance in IF birds (all *P* < 0.05), including various superpathways of menaquinol biosynthesis (all *P* < 0.01), superpathway of phylloquinol biosynthesis (*P* < 0.01), 1,4-dihydroxy-2-naphthoate biosynthesis I (*P* < 0.01), and enterobactin biosynthesis (*P* < 0.01), while 69 pathways were predicted to be in greater relative abundance in C birds (all *P* < 0.05), including superpathway of sulfur oxidation (*Acidanus amivalens*) (*P* < 0.01), succinate fermentation to butanoate (*P* < 0.01), superpathway of heme biosynthesis from glycine (*P* < 0.01), GDP-d-glycero-alpha-d-manno-heptose bioysynthesis (*P* < 0.01), and aerobic respiration I (cytochrome c) (*P* < 0.01) (Fig. 7C). In IlM microbiota, 92 pathways were predicted to be in greater relative abundance in IF birds (all *P* < 0.05), including urate biosynthesis/inosine 5ʹ-phosphate degradation (*P* < 0.01), superpathway of methylglyoxal degradation (*P* < 0.01), 3-phenylpropanoate and 3-(3-hydroxyphenyl)propanoate degradation (*P* < 0.01), cinnamate and 3-dhydroxycinnamate degradation (*P* < 0.01), and purine nucleotides degradation II (aerobic) (*P* < 0.01) (Fig. 7D), while 32 pathways were predicted to be in greater relative abundance in C birds (all *P* < 0.05), such as glutaryl-CoA degradation (*P* < 0.01) and l-glutamate and l-glutamine biosynthesis (*P* < 0.01).

## Discussion

This study aimed to determine the effects of *E. acervulina* infection on bacterial diversity, abundance, and predicted metabolic function in the luminal and mucosal microbiota of the cecum and ileum during a 14-day post-infection period. We hypothesized that *E. acervulina* infection would have differing effects on microbiota depending on the region of microbiota, as alpha and beta diversity have been shown to differ in luminal and mucosal microbiota within the cecum, ileum, jejunum, and duodenum^39^. It should be noted that our feed contained a higher crude protein percentage than usual, however, another study has shown that diets with a crude protein difference of 7% did not have significantly different alpha and beta diversity results^40^. We observed differential effects based on microbiota region in our study, where *E. acervulina* infection reduced evenness on day 10 in the CeL microbiota of IF birds, while there was no effect on evenness in the CeM microbiota of IF birds. Instead, richness was increased in CeM microbiota, with observed features affected on day 5 and Faith PD affected on day 3. The difference in time points for these two metrics may be explained by observed features being a count of unique ASVs, while Faith PD considers phylogenetic branch lengths. Overall, there appeared to be a delayed effect of infection on CeL microbiota, as microbiota became distinct between IF and C birds after the typical *E. acervulina* peak infection period of 5–7 days PI^41^. Significantly reduced plasma carotenoids in IF birds of our study on days 5 and 7 PI^38^ supported that this period was the peak of infection. CeL microbiota were distinct on day 10 and 14 based on presence and absence of ASVs (unweighted UniFrac) and distinct on day 14 based on ASV abundances (weighted UniFrac). In contrast, CeM microbiota were affected by infection at earlier points (unweighted UniFrac: day 3, weighted UniFrac: day 5) and microbiota was then affected at a later point based on presence of ASVs (unweighted UniFrac: day 14). Despite CeL and CeM microbiota sharing some dominant genera, these results show that the luminal and mucosal microbiota in the cecum are affected differently by *E. acervulina* infection and at different time points.

In the ileum, dominant taxa differed between IlL and IlM microbiota, and *E. acervulina* infection affected bacterial diversity in IlL, but not IlM, microbiota. *Lactobacillus* tended to dominate IlL microbiota from day 0 to day 10, while *Candidatus Arthromitus* tended to dominate IlM microbiota in the same time period. Between day 10 and day 14, these dominant bacteria generally decreased in relative abundance in both IlL and IlM microbiota, while *Romboutsia* greatly increased and often became dominant. *Lactobacillus* also increased to a smaller degree in IlM microbiota during this period. *E. acervulina* infection reduced richness in IlL microbiota over a relatively long period, significantly affecting observed features from day 3 to day 7 and Faith PD from day 3 to day 10. Additionally, IlL microbiota were distinct between IF and C birds based on ASV presence (but not ASV abundances) on day 3, 7, and 10, further supporting that infection affected IlL microbiota over a relatively long period. Both alpha and beta diversity were not significantly affected in IlM microbiota, again demonstrating that infection can affect one region of microbiota and not the other.

To determine genera affected by *E. acervulina* infection, we performed differential abundance analysis comparing bacterial relative abundance in IF birds and C birds. Unclassified Lachnospiraceae were in lower relative abundance in the CeL microbiota of IF birds, a similar pattern to that observed in the CeL and CeM microbiota of chickens infected by *E. tenella*^18^. Lachnospiraceae are potentially of importance in chicken GIT health, as human gut studies have demonstrated the Lachnospiraceae group contains species associated with the production of short-chain fatty acids (SCFAs) such as butyrate^42–44^. In broiler chickens, *Eimeria* infection can lead to a decrease in butyrate concentration in the cecal contents^45,46^, which likely contributes to a decrease in body weight gain, depending on the bird breed and the *Eimeria* species^47^. Butyrate supplements, such as tributyrin or sodium butyrate, have shown promise to improve body weight gain in chickens infected with *E. maxima*^48,49^, as well as in chickens with nutrient-reduced diets^50^. Although supplementation may differ in terms of concentration and how the butyrate is absorbed, it has been observed that relative abundance of Lachnospiraceae may increase^51,52^, possibly improving natural butyrate production as well. Moreover, Lachnospiraceae abundance in the cecum has been positively correlated with body weight gain including chickens infected by *E. tenella*^18^ and those challenged with both *E. maxima* and *Clostridium perfringens*^53^. Fermented soybean meal supplementation has been suggested to improve Lachnospiraceae abundance in the cecum, along with *Lachnoclostridium*, *Gastranaerophilales*, and *Lactobacillus*, and lead to improved body weight gain and food conversion ratio in broilers^54^. The genus *Butyricicoccus* and family Butyricicoccaceae were also in lower relative abundance in the CeL and CeM microbiota, respectively, of IF birds. Although 16S data cannot pinpoint species, this taxonomic group is noteworthy because *Butyricicoccus pullicaecorum*, a butyrate-producing bacterium isolated from the chicken cecum^55^, may potentially be valuable as a probiotic that improves feed conversion and contributes to prevention of necrotic enteritis^56^. Lastly, another butyrate-producing bacterium *Anaerotruncus*^57^ was in lower relative abundance in CeM microbiota of IF birds, demonstrating multiple butyrate-producing taxa may be decreased in the cecum during infection.

In contrast to decreases of Butyricicoccaceae and *Anaerotruncus* in CeM microbiota, various taxa increased in relative abundance in infected birds. Interestingly, while infection with *E. tenella* tends to increase abundance of potential secondary pathogens such as *Escherichia coli* in the cecum^18,58,59^, infection with *E. acervulina* in our study increased relative abundance of genera that potentially contain strains with beneficial functions, though further research would be required to confirm the function of these bacteria. Some of these genera have been associated with probiotic potential, such as *Bacteroides*^60,61^, *Lactobacillus*^62–64^, and *Anaerostipes*^57^, or have shown to contribute beneficial health effects, such as *Colidextribacter*^65^ and *Lachnoclostridium*^66^. While *E. tenella* directly targets the cecum and causes physical damage, *E. acervulina* primarily targets the duodenum and is less pathogenic in comparison due to smaller oocysts and fewer asexual cycles in reproduction. Therefore, lesser physical damage to the cecum may explain the difference in types of bacteria in increased abundance in IF birds. For *Lactobacillus* in particular, high crude protein % in our feed may have affected their abundance^40^, however, as feed was the same for all birds in this study, the differential abundance in C and IF birds can be attributed to infection. In IlL microbiota, results generally indicated reduced abundances of various taxa in infected birds, possibly suggesting infection made it less likely for some rarer taxa to become established, consistent with the decreases in richness observed in IlL microbiota. Of these taxa, unclassified Peptostreptococcaceae, a family with butyrate-producing members in human infants^67^, and unclassified Lachnospiraceae were identified to follow the same pattern in duodenal luminal and jejunal luminal microbiota, respectively^11^. The impact on overall butyrate production in comparison to the cecum is unclear, due to the ileum, duodenum, and jejunum containing lower absolute bacterial counts and differing in microbiota composition compared to the cecum^10^. For IlM microbiota, IF birds were more likely to contain high relative abundances of *Escherichia-Shigella* (e.g. up to 48%), while C birds typically contained less than 1%.

To search for potential links between differential abundance of taxa and functional profiles of the microbiota related to SCFA production, we utilized predictive functional analysis. Although accuracy of functional prediction can be limited outside of human studies^68^, patterns observed may assist in designing future research studies using methods such as shotgun metagenome sequencing, transcriptomics, and metabolomics. In a previous study on luminal microbiota of the duodenum and jejunum, genes predicted to influence pathways related to acetyl-CoA production were in lower relative abundance in IF birds^11^. Two major pathways utilize acetyl-CoA in butyrate production^57^, presenting the possibility that reduced butyrate production in microbiota may contribute to decreased body weight gain during infection. We observed a similar pattern in CeL microbiota, where genes predicted to affect pathways for glutaryl-CoA degradation, l-glutamate degradation V (via hydroxyglutarate), and pyruvate fermentation to butanoate (butyrate) were in lower relative abundance in IF birds. Furthermore, l-glutamate degradation V was predicted to be lower in the CeM microbiota of IF birds, and the pathways succinate fermentation to butanoate and acetyl-CoA fermentation to butanoate II were predicted to be lower in the IlL microbiota of IF birds.

## Conclusions

*Eimeria acervulina* infection affected alpha and beta diversity in the CeL, CeM, and IlL microbiota at varying time points, with CeL microbiota affected after the peak of infection, CeM microbiota affected both early and late in infection, and IlL microbiota for a more prolonged period (day 3–day 10). This study provided further evidence that *Eimeria* infections may reduce the relative abundance of potential SCFA-producing bacteria, with CeL, CeM, and IlL microbiota demonstrating this pattern. Predicted functional abundances suggest some pathways related to butyrate production could be affected as relative abundance of these bacteria are reduced during infection. Continued research to confirm the function of gut bacteria in SCFA production and determine the effectiveness of probiotics or SCFA supplementation on performance and health during *Eimeria* infections will be of importance in development of alternative solutions to antimicrobials.

### Supplementary Information

Supplementary Tables.

## Data Availability

The 16S rRNA gene sequences determined in this study were deposited in the NCBI Sequence Read Archive (SRA) database (https://www.ncbi.nlm.nih.gov/bioproject; SRA accession no. PRJNA1066775).
