# Supplementary material for: Effects of Eimeria acervulina infection on the luminal and mucosal microbiota of the cecum and ileum in broiler chickens
Source: Sci Rep. 2024 May 10;14:10702. doi: 10.1038/s41598-024-61299-6 (PMC11087572; doi:10.1038/s41598-024-61299-6)
Supplement: Supplementary file 1 — Supplementary Tables. [file 41598_2024_61299_MOESM1_ESM.docx]

Table S1. Summary table of p-values from analysis of all groups (combination of treatment and time point) using alpha and beta diversity metrics. Alpha diversity metrics (Shannon, observed features, Faith PD, and evenness) were analyzed using the Kruskal-Wallis test, while beta diversity metrics (UniFrac) were analyzed using PERMANOVA. Significant differences (p < 0.05) are bolded, and these metrics were further analyzed to compare control and infected treatments at different time points.

|  | Cecal luminal | Cecal mucosal | Ileal luminal | Ileal mucosal |
| --- | --- | --- | --- | --- |
| Shannon | **0.03** | 0.08 | 0.44 | 0.06 |
| Observed features | 0.24 | **0.02** | **<0.01** | 0.41 |
| Faith PD | **0.04** | **0.02** | **<0.01** | 0.41 |
| Evenness | **<0.01** | **0.02** | 0.57 | **0.04** |
| Unweighted UniFrac | **<0.01** | **<0.01** | **<0.01** | 0.16 |
| Weighted UniFrac | **<0.01** | **<0.01** | **<0.01** | **<0.01** |

Table S2A. P-value results from Kruskal-Wallis pairwise comparisons of Shannon diversity based on group (treatment and time) in cecal luminal microbiota. Significant differences (p < 0.05) between groups are bolded. C = Control, IF = Infected.

|  | C 0 | C 3 | C 5 | C 7 | C 10 | C 14 | IF 3 | IF 5 | IF 7 | IF 10 |
| --- | --- | --- | --- | --- | --- | --- | --- | --- | --- | --- |
| C 3 | 0.65 |  |  |  |  |  |  |  |  |  |
| C 5 | 0.10 | 0.28 |  |  |  |  |  |  |  |  |
| C 7 | 0.09 | 0.29 | 1.00 |  |  |  |  |  |  |  |
| C 10 | 0.46 | 0.51 | 0.83 | 0.72 |  |  |  |  |  |  |
| C 14 | 0.46 | 0.83 | 0.28 | 0.29 | 0.28 |  |  |  |  |  |
| IF 3 | 0.08 | 0.30 | 0.65 | 0.62 | 0.65 | 0.053 |  |  |  |  |
| IF 5 | 0.60 | 0.88 | 0.88 | 0.81 | 0.88 | 0.46 | 0.35 |  |  |  |
| IF 7 | 0.75 | 0.88 | 0.30 | 0.33 | 0.65 | 0.65 | 0.25 | 0.75 |  |  |
| IF 10 | **0.01** | 0.10 | **0.03** | **0.01** | 0.053 | 0.10 | **0.01** | **0.047** | 0.12 |  |
| IF 14 | **0.01** | 0.16 | **0.03** | **0.02** | 0.08 | 0.16 | **0.01** | 0.05 | 0.09 | 0.81 |

Table S2B. P-value results from Kruskal-Wallis pairwise comparisons of Faith’s phylogenetic diversity based on group (treatment and time) in cecal luminal microbiota. Significant differences (p < 0.05) between groups are bolded. C = Control, IF = Infected.

|  | C 0 | C 3 | C 5 | C 7 | C 10 | C 14 | IF 3 | IF 5 | IF 7 | IF 10 |
| --- | --- | --- | --- | --- | --- | --- | --- | --- | --- | --- |
| C 3 | 0.65 |  |  |  |  |  |  |  |  |  |
| C 5 | 0.30 | 0.28 |  |  |  |  |  |  |  |  |
| C 7 | 0.14 | 0.16 | 0.16 |  |  |  |  |  |  |  |
| C 10 | 0.053 | **0.0496** | 0.13 | 0.48 |  |  |  |  |  |  |
| C 14 | 0.053 | 0.13 | 0.28 | 0.48 | 0.51 |  |  |  |  |  |
| IF 3 | 0.25 | 0.18 | 0.65 | 0.33 | 0.18 | 0.30 |  |  |  |  |
| IF 5 | 0.08 | 0.053 | 0.30 | 0.81 | 0.65 | 0.88 | 0.46 |  |  |  |
| IF 7 | 0.12 | 0.18 | 0.46 | 0.62 | 0.65 | 0.65 | 0.46 | 0.92 |  |  |
| IF 10 | **0.01** | **0.03** | **0.03** | 0.09 | 0.46 | 0.30 | **0.01** | 0.17 | **0.01** |  |
| IF 14 | 0.09 | 0.08 | 0.48 | 0.77 | 0.72 | 1.00 | 0.22 | 0.81 | 0.46 | 0.22 |

Table S2C. P-value results from Kruskal-Wallis pairwise comparisons of evenness based on group (treatment and time) in cecal luminal microbiota. Significant differences (p < 0.05) between groups are bolded. C = Control, IF = Infected.

|  | C 0 | C 3 | C 5 | C 7 | C 10 | C 14 | IF 3 | IF 5 | IF 7 | IF 10 |
| --- | --- | --- | --- | --- | --- | --- | --- | --- | --- | --- |
| C 3 | 0.88 |  |  |  |  |  |  |  |  |  |
| C 5 | 0.053 | **0.0496** |  |  |  |  |  |  |  |  |
| C 7 | 0.33 | 0.48 | **0.03** |  |  |  |  |  |  |  |
| C 10 | 0.88 | 0.83 | 0.51 | 0.48 |  |  |  |  |  |  |
| C 14 | **0.03** | 0.28 | **0.0496** | **0.03** | 0.28 |  |  |  |  |  |
| IF 3 | **0.047** | 0.10 | 0.46 | 0.09 | 0.30 | **0.03** |  |  |  |  |
| IF 5 | 0.75 | 0.65 | 0.18 | 1.00 | 0.88 | 0.18 | 0.35 |  |  |  |
| IF 7 | 0.46 | 0.88 | 0.10 | 0.33 | 0.46 | 0.65 | 0.08 | 0.35 |  |  |
| IF 10 | **0.01** | **0.03** | **0.03** | **0.01** | **0.03** | 0.10 | **0.01** | **0.03** | **0.047** |  |
| IF 14 | **0.01** | 0.08 | **0.03** | **0.02** | 0.08 | 0.29 | **0.01** | 0.05 | 0.09 | 0.81 |

Table S2D. P-value results from PERMANOVA pairwise comparisons of unweighted UniFrac based on group (treatment and time) in cecal luminal microbiota. Significant differences (p < 0.05) between groups are bolded. C = Control, IF = Infected.

|  | C 0 | C 3 | C 5 | C 7 | C 10 | C 14 | IF 3 | IF 5 | IF 7 | IF 10 |
| --- | --- | --- | --- | --- | --- | --- | --- | --- | --- | --- |
| C 3 | 0.07 |  |  |  |  |  |  |  |  |  |
| C 5 | **0.02** | 0.10 |  |  |  |  |  |  |  |  |
| C 7 | **0.01** | 0.08 | 0.60 |  |  |  |  |  |  |  |
| C 10 | **0.02** | 0.09 | 0.50 | 0.37 |  |  |  |  |  |  |
| C 14 | **0.02** | 0.09 | 0.11 | **0.03** | 0.20 |  |  |  |  |  |
| IF 3 | **0.01** | 0.89 | 0.37 | 0.29 | **0.04** | **0.02** |  |  |  |  |
| IF 5 | **0.01** | 0.23 | 0.38 | 0.06 | 0.15 | **0.05** | 0.07 |  |  |  |
| IF 7 | **0.01** | 0.10 | 0.53 | 0.24 | 0.45 | **0.04** | 0.10 | 0.21 |  |  |
| IF 10 | **0.01** | **0.02** | **0.02** | **0.01** | **0.02** | **0.01** | **0.01** | **0.01** | **0.01** |  |
| IF 14 | **0.01** | **0.03** | **0.04** | **0.03** | **0.03** | **0.05** | **0.01** | **0.00** | **0.01** | 0.11 |

Table S2E. P-value results from PERMANOVA pairwise comparisons of weighted UniFrac based on group (treatment and time) in cecal luminal microbiota. Significant differences (p < 0.05) between groups are bolded. C = Control, IF = Infected.

|  | C 0 | C 3 | C 5 | C 7 | C 10 | C 14 | IF 3 | IF 5 | IF 7 | IF 10 |
| --- | --- | --- | --- | --- | --- | --- | --- | --- | --- | --- |
| C 3 | 0.10 |  |  |  |  |  |  |  |  |  |
| C 5 | 0.16 | 0.29 |  |  |  |  |  |  |  |  |
| C 7 | **0.01** | 0.15 | 0.15 |  |  |  |  |  |  |  |
| C 10 | **0.02** | 0.19 | 0.29 | 0.17 |  |  |  |  |  |  |
| C 14 | **0.02** | 0.10 | 0.09 | 0.09 | 0.33 |  |  |  |  |  |
| IF 3 | 0.06 | 0.44 | 0.83 | 0.17 | 0.07 | **0.02** |  |  |  |  |
| IF 5 | **0.05** | 0.34 | 0.33 | 0.051 | 0.09 | **0.02** | 0.58 |  |  |  |
| IF 7 | **0.01** | 0.21 | 0.16 | 0.08 | 0.07 | 0.10 | 0.15 | 0.14 |  |  |
| IF 10 | **0.00** | 0.12 | 0.15 | 0.18 | 0.21 | 0.16 | 0.06 | 0.10 | **0.01** |  |
| IF 14 | **0.01** | 0.053 | 0.07 | **0.02** | 0.06 | 0.057 | **0.01** | **0.01** | **0.01** | 0.18 |

Table S3A. P-value results from Kruskal-Wallis pairwise comparisons of observed features based on group (treatment and time) in cecal mucosal microbiota. Significant differences (p < 0.05) between groups are bolded. C = Control, IF = Infected.

|  | C 0 | C 3 | C 5 | C 7 | C 10 | C 14 | IF 3 | IF 5 | IF 7 | IF 10 |
| --- | --- | --- | --- | --- | --- | --- | --- | --- | --- | --- |
| C 3 | 1.00 |  |  |  |  |  |  |  |  |  |
| C 5 | 0.88 | 0.72 |  |  |  |  |  |  |  |  |
| C 7 | 0.33 | 0.47 | 0.29 |  |  |  |  |  |  |  |
| C 10 | 0.07 | 0.16 | 0.13 | 0.72 |  |  |  |  |  |  |
| C 14 | **0.01** | **0.02** | **0.03** | 0.15 | 0.72 |  |  |  |  |  |
| IF 3 | 0.08 | 0.09 | 0.10 | 1.00 | 0.65 | 0.07 |  |  |  |  |
| IF 5 | **0.03** | **0.049** | **0.03** | 0.81 | 0.88 | 0.14 | 0.75 |  |  |  |
| IF 7 | **0.01** | **0.01** | **0.03** | 0.71 | 1.00 | 0.09 | 0.83 | 0.92 |  |  |
| IF 10 | **0.02** | **0.02** | **0.02** | 0.62 | 0.88 | 0.22 | 0.40 | 0.92 | 0.75 |  |
| IF 14 | 0.06 | 0.05 | 0.052 | 0.81 | 0.46 | 0.05 | 0.75 | 0.67 | 0.25 | 0.35 |

Table S3B. P-value results from Kruskal-Wallis pairwise comparisons of Faith’s phylogenetic diversity based on group (treatment and time) in cecal mucosal microbiota. Significant differences (p < 0.05) between groups are bolded. C = Control, IF = Infected.

|  | C 0 | C 3 | C 5 | C 7 | C 10 | C 14 | IF 3 | IF 5 | IF 7 | IF 10 |
| --- | --- | --- | --- | --- | --- | --- | --- | --- | --- | --- |
| C 3 | 0.62 |  |  |  |  |  |  |  |  |  |
| C 5 | 0.88 | 0.72 |  |  |  |  |  |  |  |  |
| C 7 | **0.03** | 0.08 | 0.16 |  |  |  |  |  |  |  |
| C 10 | 0.053 | 0.08 | 0.13 | 1.00 |  |  |  |  |  |  |
| C 14 | 0.62 | 1.00 | 0.72 | 0.15 | 0.16 |  |  |  |  |  |
| IF 3 | **0.01** | **0.01** | **0.03** | 0.62 | 0.46 | 0.01 |  |  |  |  |
| IF 5 | 0.12 | 0.09 | 0.10 | 0.62 | 0.30 | 0.14 | 0.25 |  |  |  |
| IF 7 | 0.12 | 0.22 | 0.46 | 0.46 | 0.30 | 0.22 | **0.02** | 0.35 |  |  |
| IF 10 | 0.60 | 0.46 | 0.88 | 0.14 | 0.18 | 0.62 | **0.02** | 0.08 | 0.25 |  |
| IF 14 | **0.03** | 0.09 | 0.10 | 0.62 | 0.46 | 0.14 | 0.17 | 0.75 | 0.46 | 0.12 |

Table S3C. P-value results from Kruskal-Wallis pairwise comparisons of evenness based on group (treatment and time) in cecal mucosal microbiota. Significant differences (p < 0.05) between groups are bolded. C = Control, IF = Infected.

|  | C 0 | C 3 | C 5 | C 7 | C 10 | C 14 | IF 3 | IF 5 | IF 7 | IF 10 |
| --- | --- | --- | --- | --- | --- | --- | --- | --- | --- | --- |
| C 3 | 0.05 |  |  |  |  |  |  |  |  |  |
| C 5 | 0.053 | 0.72 |  |  |  |  |  |  |  |  |
| C 7 | 0.22 | 0.77 | 0.72 |  |  |  |  |  |  |  |
| C 10 | **0.03** | 0.29 | 0.28 | 0.72 |  |  |  |  |  |  |
| C 14 | **0.01** | 0.15 | 0.29 | 0.56 | 1.00 |  |  |  |  |  |
| IF 3 | **0.047** | 0.81 | 0.46 | 0.81 | 0.30 | 0.14 |  |  |  |  |
| IF 5 | 0.25 | 0.03 | 0.053 | 0.14 | 0.03 | 0.01 | **0.047** |  |  |  |
| IF 7 | 0.92 | 0.14 | 0.18 | 0.33 | 0.10 | 0.09 | 0.12 | 0.46 |  |  |
| IF 10 | 0.17 | 0.81 | 0.65 | 0.81 | 0.46 | 0.62 | 0.75 | 0.08 | 0.12 |  |
| IF 14 | **0.01** | 0.05 | 0.10 | 0.46 | 0.46 | 0.46 | 0.08 | **0.01** | **0.047** | 0.60 |

Table S3D. P-value results from PERMANOVA pairwise comparisons of unweighted UniFrac based on group (treatment and time) in cecal mucosal microbiota. Significant differences (p < 0.05) between groups are bolded. C = Control, IF = Infected.

|  | C 0 | C 3 | C 5 | C 7 | C 10 | C 14 | IF 3 | IF 5 | IF 7 | IF 10 |
| --- | --- | --- | --- | --- | --- | --- | --- | --- | --- | --- |
| C 3 | 0.66 |  |  |  |  |  |  |  |  |  |
| C 5 | **0.03** | 0.06 |  |  |  |  |  |  |  |  |
| C 7 | **0.02** | 0.16 | 0.12 |  |  |  |  |  |  |  |
| C 10 | **0.02** | 0.13 | 0.12 | 0.95 |  |  |  |  |  |  |
| C 14 | **0.04** | 0.29 | **0.03** | 0.052 | 0.10 |  |  |  |  |  |
| IF 3 | **0.01** | **0.04** | **0.02** | 0.62 | 0.78 | **0.01** |  |  |  |  |
| IF 5 | 0.10 | 0.19 | 0.17 | 0.68 | 0.44 | 0.08 | 0.16 |  |  |  |
| IF 7 | **0.02** | 0.15 | **0.02** | 0.20 | 0.11 | 0.20 | **0.01** | 0.36 |  |  |
| IF 10 | 0.07 | 0.23 | **0.02** | 0.09 | 0.08 | 0.20 | **0.02** | 0.06 | 0.08 |  |
| IF 14 | **0.02** | **0.03** | **0.01** | 0.10 | 0.12 | 0.01 | 0.30 | 0.13 | **0.01** | **0.03** |

Table S3E. P-value results from PERMANOVA pairwise comparisons of weighted UniFrac based on group (treatment and time) in cecal mucosal microbiota. Significant differences (p < 0.05) between groups are bolded. C = Control, IF = Infected.

|  | C 0 | C 3 | C 5 | C 7 | C 10 | C 14 | IF 3 | IF 5 | IF 7 | IF 10 |
| --- | --- | --- | --- | --- | --- | --- | --- | --- | --- | --- |
| C 3 | **0.04** |  |  |  |  |  |  |  |  |  |
| C 5 | **0.02** | 0.26 |  |  |  |  |  |  |  |  |
| C 7 | 0.10 | 0.66 | 0.54 |  |  |  |  |  |  |  |
| C 10 | **0.02** | 0.17 | 0.68 | 0.57 |  |  |  |  |  |  |
| C 14 | **0.01** | 0.051 | 0.13 | 0.06 | 0.53 |  |  |  |  |  |
| IF 3 | 0.11 | 0.79 | 0.32 | 0.62 | 0.17 | 0.08 |  |  |  |  |
| IF 5 | 0.06 | 0.10 | **0.03** | 0.19 | **0.02** | **0.01** | 0.42 |  |  |  |
| IF 7 | **0.04** | **0.03** | **0.02** | 0.17 | **0.01** | **0.01** | 0.14 | 0.20 |  |  |
| IF 10 | **0.03** | 0.37 | 0.31 | 0.38 | 0.26 | 0.30 | 0.35 | 0.28 | 0.12 |  |
| IF 14 | **0.01** | **0.05** | 0.13 | 0.06 | 0.24 | 0.10 | **0.03** | **<0.01** | **0.01** | 0.38 |

Table S4A. P-value results from Kruskal-Wallis pairwise comparisons of observed features based on group (treatment and time) in ileal luminal microbiota. Significant differences (p < 0.05) between groups are bolded. C = Control, IF = Infected.

|  | C 0 | C 3 | C 5 | C 7 | C 10 | C 14 | IF 3 | IF 5 | IF 7 | IF 10 |
| --- | --- | --- | --- | --- | --- | --- | --- | --- | --- | --- |
| C 3 | 0.46 |  |  |  |  |  |  |  |  |  |
| C 5 | **0.03** | **0.03** |  |  |  |  |  |  |  |  |
| C 7 | 0.62 | 0.25 | **0.03** |  |  |  |  |  |  |  |
| C 10 | 0.88 | 0.72 | **0.0495** | 0.48 |  |  |  |  |  |  |
| C 14 | **0.01** | 0.08 | 0.86 | **0.02** | **0.0497** |  |  |  |  |  |
| IF 3 | **0.01** | **0.03** | 0.30 | **0.01** | **0.03** | 0.62 |  |  |  |  |
| IF 5 | **0.01** | **0.02** | **0.03** | **0.02** | **0.03** | **0.03** | 0.05 |  |  |  |
| IF 7 | **0.01** | **0.01** | **0.03** | **0.01** | **0.03** | **0.01** | **0.02** | 0.81 |  |  |
| IF 10 | **0.01** | 0.09 | 0.23 | **0.01** | 0.053 | 0.62 | 0.35 | 0.11 | 0.12 |  |
| IF 14 | **0.01** | **0.04** | 0.16 | **0.02** | **0.03** | 1.00 | 0.62 | **0.02** | **0.01** | 0.33 |

Table S4B. P-value results from Kruskal-Wallis pairwise comparisons of Faith’s phylogenetic diversity based on group (treatment and time) in ileal luminal microbiota. Significant differences (p < 0.05) between groups are bolded. C = Control, IF = Infected.

|  | C 0 | C 3 | C 5 | C 7 | C 10 | C 14 | IF 3 | IF 5 | IF 7 | IF 10 |
| --- | --- | --- | --- | --- | --- | --- | --- | --- | --- | --- |
| C 3 | 0.33 |  |  |  |  |  |  |  |  |  |
| C 5 | **0.03** | **0.03** |  |  |  |  |  |  |  |  |
| C 7 | 0.81 | 0.25 | **0.03** |  |  |  |  |  |  |  |
| C 10 | 0.65 | 0.72 | **0.0495** | 0.48 |  |  |  |  |  |  |
| C 14 | **0.01** | **0.02** | 0.48 | **0.02** | **0.03** |  |  |  |  |  |
| IF 3 | **0.01** | **0.01** | 0.30 | **0.01** | **0.03** | 0.33 |  |  |  |  |
| IF 5 | **0.01** | **0.02** | **0.03** | **0.02** | **0.03** | 0.25 | **0.01** |  |  |  |
| IF 7 | **0.047** | **0.03** | 0.18 | **0.03** | 0.053 | 0.46 | 0.12 | 0.62 |  |  |
| IF 10 | **0.01** | **0.03** | 0.65 | **0.01** | **0.03** | 0.33 | 0.60 | 0.14 | 0.25 |  |
| IF 14 | **0.01** | **0.02** | **0.03** | **0.02** | **0.03** | 0.77 | **0.03** | 0.56 | 0.33 | 0.14 |

Table S4C. P-value results from PERMANOVA pairwise comparisons of unweighted UniFrac based on group (treatment and time) in ileal luminal microbiota. Significant differences (p < 0.05) between groups are bolded. C = Control, IF = Infected.

|  | C 0 | C 3 | C 5 | C 7 | C 10 | C 14 | IF 3 | IF 5 | IF 7 | IF 10 |
| --- | --- | --- | --- | --- | --- | --- | --- | --- | --- | --- |
| C 3 | 0.46 |  |  |  |  |  |  |  |  |  |
| C 5 | **0.02** | 0.05 |  |  |  |  |  |  |  |  |
| C 7 | 0.09 | 0.12 | **0.04** |  |  |  |  |  |  |  |
| C 10 | 0.21 | 0.61 | 0.12 | **0.02** |  |  |  |  |  |  |
| C 14 | **0.02** | 0.06 | 0.36 | **0.02** | 0.06 |  |  |  |  |  |
| IF 3 | **0.01** | **0.01** | 0.21 | **0.01** | **0.02** | 0.06 |  |  |  |  |
| IF 5 | **0.01** | **0.04** | 0.055 | **0.02** | **0.02** | 0.12 | **0.01** |  |  |  |
| IF 7 | **0.01** | **0.01** | **0.04** | **0.01** | **0.03** | 0.38 | **0.01** | 0.12 |  |  |
| IF 10 | **0.01** | **0.02** | 0.78 | **0.01** | **0.02** | 0.52 | 0.19 | 0.06 | **0.03** |  |
| IF 14 | **0.01** | **0.03** | **0.03** | **0.02** | **0.03** | 0.98 | **0.01** | **0.02** | **0.04** | 0.06 |

Table S4D. P-value results from PERMANOVA pairwise comparisons of weighted UniFrac based on group (treatment and time) in ileal luminal microbiota. Significant differences (p < 0.05) between groups are bolded. C = Control, IF = Infected.

|  | C 0 | C 3 | C 5 | C 7 | C 10 | C 14 | IF 3 | IF 5 | IF 7 | IF 10 |
| --- | --- | --- | --- | --- | --- | --- | --- | --- | --- | --- |
| C 3 | 0.60 |  |  |  |  |  |  |  |  |  |
| C 5 | 0.18 | 0.29 |  |  |  |  |  |  |  |  |
| C 7 | 0.30 | 0.26 | 0.46 |  |  |  |  |  |  |  |
| C 10 | 0.47 | 0.79 | 0.90 | 0.73 |  |  |  |  |  |  |
| C 14 | 0.17 | 0.09 | 0.40 | 0.08 | 0.36 |  |  |  |  |  |
| IF 3 | 0.07 | 0.056 | 0.36 | 0.85 | 0.49 | 0.10 |  |  |  |  |
| IF 5 | 0.06 | **0.03** | 0.13 | 0.21 | 0.10 | 0.06 | 0.26 |  |  |  |
| IF 7 | 0.06 | 0.07 | 0.39 | 0.63 | 0.24 | 0.16 | 0.48 | 0.26 |  |  |
| IF 10 | 0.69 | 0.42 | 0.67 | 0.64 | 0.77 | 0.77 | 0.74 | 0.29 | 0.33 |  |
| IF 14 | 0.09 | **0.03** | 0.17 | **0.02** | 0.08 | 0.67 | **0.05** | 0.05 | 0.09 | 0.49 |

Table S5A. P-value results from Kruskal-Wallis pairwise comparisons of evenness based on group (treatment and time) in ileal mucosal microbiota. Significant differences (p < 0.05) between groups are bolded. C = Control, IF = Infected.

|  | C 0 | C 3 | C 5 | C 7 | C 10 | C 14 | IF 3 | IF 5 | IF 7 | IF 10 |
| --- | --- | --- | --- | --- | --- | --- | --- | --- | --- | --- |
| C 3 | 0.33 |  |  |  |  |  |  |  |  |  |
| C 5 | 0.46 | **0.03** |  |  |  |  |  |  |  |  |
| C 7 | 0.46 | 0.15 | 1.00 |  |  |  |  |  |  |  |
| C 10 | 0.65 | 0.29 | 0.83 | 1.00 |  |  |  |  |  |  |
| C 14 | 0.053 | 0.06 | 0.25 | 0.35 | 0.56 |  |  |  |  |  |
| IF 3 | 0.25 | 0.09 | 0.88 | 0.81 | 0.88 | 0.053 |  |  |  |  |
| IF 5 | 0.05 | **0.02** | 0.48 | 0.25 | 0.72 | 0.06 | 0.46 |  |  |  |
| IF 7 | 0.08 | **0.01** | 0.30 | 0.22 | 0.88 | 0.25 | 0.60 | 1.00 |  |  |
| IF 10 | 0.25 | 0.09 | 0.88 | 0.62 | 0.88 | 0.053 | 0.92 | 0.46 | 0.35 |  |
| IF 14 | **0.01** | **0.02** | **0.03** | 0.25 | 0.48 | 0.35 | **0.01** | **0.02** | 0.09 | **0.01** |

Table S5B. P-value results from PERMANOVA pairwise comparisons of weighted UniFrac based on group (treatment and time) in ileal mucosal microbiota. Significant differences (p < 0.05) between groups are bolded. C = Control, IF = Infected.

|  | C 0 | C 3 | C 5 | C 7 | C 10 | C 14 | IF 3 | IF 5 | IF 7 | IF 10 |
| --- | --- | --- | --- | --- | --- | --- | --- | --- | --- | --- |
| C 3 | 0.58 |  |  |  |  |  |  |  |  |  |
| C 5 | 0.12 | 0.04 |  |  |  |  |  |  |  |  |
| C 7 | 0.81 | 1.00 | 0.87 |  |  |  |  |  |  |  |
| C 10 | 0.19 | 0.14 | 0.81 | 0.85 |  |  |  |  |  |  |
| C 14 | 0.053 | 0.07 | 0.10 | 0.07 | 0.19 |  |  |  |  |  |
| IF 3 | 0.23 | 0.26 | 0.82 | 0.32 | 0.62 | 0.07 |  |  |  |  |
| IF 5 | 0.06 | **0.02** | 0.23 | 0.55 | 0.60 | 0.06 | 0.41 |  |  |  |
| IF 7 | **0.02** | **0.04** | 0.56 | 0.52 | 0.91 | 0.17 | 0.21 | 0.99 |  |  |
| IF 10 | 0.36 | 0.27 | 0.60 | 0.94 | 0.66 | **0.04** | 0.27 | 0.33 | 0.47 |  |
| IF 14 | **0.01** | **0.03** | **0.04** | **0.03** | **0.03** | 0.59 | **0.01** | **0.03** | **0.02** | **0.02** |
